# Impact of Antimicrobial Carcass Washes and Processing Techniques on Quality Attributes of Beef Frankfurters

**DOI:** 10.3390/foods11131891

**Published:** 2022-06-26

**Authors:** Emily Ford, Melissa Davis, Yuan H. Brad Kim, Tom Katen, Stacy M. S. Zuelly

**Affiliations:** 1 Department of Animal Science, Purdue University, West Lafayette, IN 47907, USA; ebedwell@purdue.edu (E.F.); davis565@purdue.edu (M.D.); bradkim@purdue.edu (Y.H.B.K.); 2 Cargill, Willshire, OH 45898, USA; tom_katen@cargill.com

**Keywords:** beef, frankfurter, antimicrobial carcass wash, beef trim, emulsion stability, color, sensory analysis

## Abstract

The objective of this study was to determine the impact of antimicrobial carcass washes on beef trim in the production of frankfurters. Twenty-four beef carcasses had different antimicrobial wash treatments (TRTs) randomly applied during the harvest procedure: 82 °C water (CON), peroxyacetic acid (PAA), or lactic acid (LA). Frankfurters were produced using carcass trim at two different batter temperature processes (PROC): 4 °C (LTP) and 21 °C (HTP). Frankfurters were analyzed for processing yield (PY), emulsion stability (ES), instrumental external and internal color (CIE L*, a*, b*), purge loss, texture, and sensory analysis. TRT had very little impact on frankfurter characteristics with a difference found in ES water and instrumental hardness (*p* < 0.05). PROC impacted ES water, ES fat, PY, instrumental springiness, external and internal color, sensory hardness, cohesion, and juiciness (*p* < 0.05). However, no TRT × PROC interactions were found to be significant (*p* > 0.05). These data indicate that antimicrobial carcass washes had little impact on frankfurter quality, while the processing technique impacted several frankfurter quality characteristics. This indicates that processors can impact frankfurter composition via processing techniques without concern of antimicrobial washes influencing frankfurter quality.

## 1. Introduction

Lean beef trimmings recovered during the fabrication process are utilized in many further processed products, including emulsified products, such as frankfurters. During frankfurter production, the goal is to produce a stable meat emulsion and control factors such as pH and protein extraction in order to produce a quality product in appearance and sensory characteristics [1].

The properties of pH, salt concentration, protein extraction and other non-meat ingredients all affect several sensory factors of frankfurters. When the pH of meat is further away from 5.0–5.2 (isoelectric point of meat 5.0–5.2), there are more protein side chain charges available for water binding, therefore increasing the water protein interactions and the functionality of the raw materials for further processing [2,3]. However, meat pH can cause a negative effect on emulsion stability, as proteins denature at pH values below 5.0, which will cause proteins to become ineffective in forming an emulsion [2]. Salt is necessary in frankfurters, as salt-soluble myofibular protein distribution properties increase water-binding ability and textural properties, which directly affect the gel structure formed during an emulsion, with myofibular proteins acting as primary emulsifiers and stabilizers of the meat batter [4]. Therefore, increasing the functionality of the raw materials can produce a more stable meat batter emulsion, thus producing a higher quality frankfurter [1].

Frankfurter quality is also dependent on the temperature of processing conditions, chopping time and final batter temperature [1]. Increasing meat batter temperature is critical when forming a stable emulsion, because as the batter temperature increases, fat droplets decrease in size, causing improved protein and fat interactions within the meat emulsion matrix [5]. Jones and Mandigo [5] studied the effects of endpoint chopping temperatures of 10, 16, 22, and 28 °C on frankfurters and concluded a higher cook loss of meat batters greater than 22 °C, which was caused by an emulsion breakdown when batter temperatures are too high. Colder chopping temperatures were evaluated by Sutton et al. [6], reporting endpoint chopping temperatures at a peak of 15 °C and below produced the most stable product in reduced fat high moisture beef frankfurters. Although previous research has found differences in frankfurter quality due to processing temperature, these works have all utilized consistent beef trim sources without evaluating differences in pH due to antimicrobial treatments.

To meet USDA zero-tolerance standards for food-borne pathogens, acidic antimicrobial washes are applied during harvest to decrease the microbial load of the beef carcass. Pathogenic microbes present on the carcass harbored during the harvest process are killed by antimicrobial washes at low pH [7]. Organic acids such as lactic acid and peroxyacetic acid are widely used throughout the beef industry as carcass antimicrobial agents and have been found to be extremely effective in reducing microbial contamination on beef carcasses [8,9]. Several researchers have found an immediate decrease in the surface pH of beef trim when antimicrobial washes are applied, although after days of storage, the pH equilibrated to show no difference from controls [9,10,11,12]. However, antimicrobial wash applications to beef carcasses have not been evaluated to determine the impact to superficial muscle pH and the possible subsequent effect emulsion stability and frankfurter quality.

We hypothesize carcasses treated with lower pH antimicrobial washes will have an effect of emulsion stability causing excess water and fat leakage from meat batters, in turn affecting internal, external and frankfurter sensory attributes. The objective of this study is to determine the effect of carcass antimicrobial washes and processing temperature variations on quality attributes of formulated beef frankfurters.

## 2. Materials and Methods

### 2.1. Sample Preparation

Twenty-four beef carcasses were selected from the Purdue University Land O’ Lakes Center (West Lafayette, IN, USA), over two harvest days, twelve carcasses per harvest day. After evisceration and splitting, a zero-tolerance evaluation for foreign material was completed, and carcasses were rinsed with water and allowed to drip for 5 min. Carcasses were randomly assigned to an antimicrobial carcass wash treatment (TRT), which included: 82 °C water (CON), peroxyacetic acid (PAA) or lactic acid (LAC). The PAA treatment (Crimson Chemical, Fort Worth, TX, USA) had an initial concentration of 23.72% acid solution, which was diluted to 350 ppm with water, and 1.9 L was applied to each carcass at 55 °C. The lactic acid (LAC) wash (Corbion, Lenexa, KS, USA) had an initial concentration of 88% lactic acid was diluted to 5% dilution of the acid solution and 3.8 L of per carcass at 55 °C. The washes were applied using methods prescribed for small processing plants by Cutter [13]. The acid washes were applied using a handheld sprayer approximately 30 cm in distance, applying from the posterior to anterior of the carcass at 12–15 psi. Carcasses were allowed to drip for 1 min before entering the cooler at 4 °C for 24 h. Pre-wash pH was measured with a pH probe meter (Hanna Instrument, Inc., Warner, NH, USA) in duplicate inserted superficially into the hindquarters (bicepts femoris) before carcass wash interventions (Pre-Wash pH), as well as 30 min post-wash and 24 h post-wash.

### 2.2. Frankfurter Sample Preparation

Beef lean trim (90% lean) and fat trim was collected 24 h post-harvest from the round, chuck and brisket regions of each carcass. Lean and fat trim were vacuum-packaged separately and stored at 4 °C for five days to simulate industry processing protocols. Beef trim from each side was assigned to a different frankfurter processing technique (PROC). Carcass trim from the right side of the carcass was designated for high-temperature processing (HTP), and the left side of the carcass was designated for the low-temperature processing (LTP). All trim was pre-ground 24 h before processing using a 4.76 mm grinder plate and allocated to 6.4 kg batches for each frankfurter processing method (Table 1).

For HTP frankfurters, trim was ground through a 4.76 mm plate meat grinder, and the ground trim and cure ingredients (6.25% sodium nitrite) were added to a Stephan vertical chopper (Stephan, Columbus, OH, USA) along with salt and phosphate ingredients on low speed for 1 min (Table 1). Ground fat was added and mixed on low speed for 30 s. The remaining dry non-meat ingredients and one-third of the water (337 g) were included and mixed for ten seconds. The remaining water was added (337 g), and the batter continued to mix on high for one minute to give a final batter temperature of 21 °C. Samples were collected for emulsion stability and pH analysis. The batter was vacuum-sealed using a Promarks packager (Promarks Inc., Claremont, CA, USA) to release any air from the batter.

For LTP frankfurters, a Kodiak Varimixer (Varimixer, Charlotte, NC, USA) was used with the ingredients shown in Table 1. A meat pre-blend was made with ground beef and fat mixed with the cure ingredients (6.25% sodium nitrite, A.C. Legg, Inc. Calera, AL, USA) and salt 12 h before frankfurter production for optimum protein extraction. The pre-blend fat and one-third of the initial water (377 g) was added to the frankfurter preparation and combined for 30 s. Then, the spice mix, other non-meat dry ingredients and one-third of the water (377 g) was added and mixed for 30 s. Finally, the last third of the water (377 g) was added and mixed for 30 s for a total mix time of 1.5 min. The batter was then ground twice using a 4.76 mm grind-plate reaching a final batter temperature of 4 °C. Samples were collected for emulsion stability and pH analysis. The batter was vacuum-sealed using a Promarks packager (Promarks Inc., Claremont, CA, USA) to release any air from the batter.

All meat batters were transferred into a Talsa Stuffer (Talsa, Valencia, Spain) and stuffed into a 29 mm cellulose casing. The frankfurters were then linked and weighed before the cook process (Raw Weight). The frankfurters were placed in a Scott Pec smokehouse (ScottPec, Inc Guelph, ON, Canada), cooked to an internal temperature of 71 °C, and cool-showered for 10 min before the cook weight was recorded (Final Cooked Weight). Frankfurters were held at 4 °C for approximately 12 h, at which time the casings were removed, and frankfurters were vacuum-packaged. The samples were held at 4 °C for instrumental texture, purge, and instrumental color analysis. Frankfurter sensory and extra samples were kept frozen at −40 °C until further analyzed.

Processing yield (PY) was determined by the following equation: % PY = (Final Cooked Weight/Raw Weight) × 100.

### 2.3. Frankfurter Batter pH

The pH of the fresh batter (Fresh Batter pH) was recorded approximately 6 h post processing following the procedure described by Sebranek et al. [14]. The pH samples were prepared by adding 10 g of batter to 90 mL of distilled, deionized water and homogenized for 45 s; then, it was filtered using Whatman No. 1 filter paper. A bench-top pH meter (Satorious AG, Gottingen, Germany) was calibrated to pH standards 4.0, 7.0, and 10.0 and then used to measure the sample pH in duplicate of each batter.

### 2.4. Emulsion Stability

Emulsion stability (ES) measurements were taken according to Sebranic et al. [14]. Approximately 25 g of meat batter was injected in a Wierbicki centrifuge tube and weighed (Fresh Sample Weight). The tubes were cooked in a water bath to 71 °C for 30 min, cooled for 5 min and then centrifuged at 750 gmax for six min at 25 °C. The amount of separated fat (Expelled Fat) and water (Expelled Water) expelled from each sample was recorded in mL in order to calculate the amount of fat and water that was lost during cooking. The calculation to determine ES Water Separation was: ES Water % = (mL Expelled Water/Fresh Sample Weight) × 100. The calculation to determine ES Fat Separation was: ES Fat % = (mL Expelled fat/Fresh Sample Weight) × 100.

### 2.5. Instrumental Color

Frankfurter external surface and internal color measurements were taken 24 h post processing (0 d) randomly selecting two frankfurters per package. External measurements were taken in triplicate on the outside surface of two fully cooked frankfurters. Internal color measurements were taken by cutting two frankfurters lengthwise and measuring the surface color in triplicate. Commission internationale de l’éclairage (CIE) L*, a*, and b* values were obtained using a CR-400 Chroma Meter (Konica Minolta, Chiyoda, Tokyo, Japan) equipped with a CIE standard illuminant C [15] with an 8 mm illumination area, which was calibrated using a standardized white plate. For shelf life color analysis, the frankfurters were placed under display lighting at 4 °C, and color was measured at 30 and 60 days.

### 2.6. Instrumental Texture Analysis

The frankfurters were analyzed for texture using the methods of Bourne [16] and Wenther [17]. Four frankfurter samples were prepared by cutting into two cores (1.6 cm × 1.9 cm) per frankfurter. The cores were analyzed by a TA-XT Plus Texture Analyzer (Stable Micro System Ltd., Godalming, UK) using a TA-25 cylinder probe to measure hardness, springiness, and cohesiveness.

### 2.7. Purge Loss

Purge loss (PL) frankfurters were measured 2 weeks post processing, using packages with 6 frankfurters that had been held at 4 °C. The total weight of the package with frankfurters was recorded (Total Package Weight), packages were then opened, frankfurters were blotted and weighed (Frankfurter Weight), and the dry bag was weighed (Bag Weight). Total PL was calculated using the following equation: % PL = ((Total Package Weight − Frankfurter Weight − Bag Weight)/Total Package Weigh) × 100.

### 2.8. Sensory Analysis

Sensory analysis was performed using a trained panel with a minimum of 8 panelists per setting. The panelist performed 1 h training sessions to familiarize themselves with the sensory characteristics using 6 different types of commercially made frankfurters. The frankfurters were analyzed using an 8-point hedonic scale for each sensory attribute of hardness (1 = extremely hard, 8 = extremely soft), cohesiveness (1 = extremely dissolves, 8 = extremely cohesive), juiciness (1 = extremely dry, 8 = extremely juicy), flavor intensity 1 = extremely bland, 8 = extremely flavorful), and off flavor (1 = extremely intense, 8 = non detectable).

The frankfurter samples were prepared in a water bath until an internal temperature of 71 °C was reached. The panelists were given two 1 cm sliced samples for each frankfurter under red lighting in the sensory booths. The panelists cleansed their palate using unsalted saltine crackers and water between each sample. The panelists were given 6 samples to evaluate with an equal representation of carcass wash treatments (CON, PAA, and LAC) and frankfurter processes (LTP and HTP).

### 2.9. Statistical Analysis

The experimental design was a 3 × 2 structure in a randomized complete block design of TRT and PROC with carcass side as the experimental unit (*n* = 48). The 3 TRT (CON, LA, PAA) were applied to 12 carcasses and replicated over 2 harvest days (*n* = 24). Each carcass was slit down the medial line, and trim from each side was allocated to the two PROC of LTP and HTP. The fixed main effects of TRT and PROC, the random effect of harvest day, and their interactions were analyzed using the mixed procedure SAS software package (SAS version 9.4; SAS Institute Inc., Cary, NC, USA, 2012) for ANOVA of microbial analysis, emulsion stability, texture analysis, batter pH, carcass pH, processing yield, purse loss, and sensory analysis. The fixed main effects of TRT, PROC, and display day, the random effect of harvest day, and all subsequent interaction were also analyzed using the mixed procedure SAS software for ANOVA of instrumental color. Least squares means for all traits were separated by using least significant differences analyzed by the PDIFF option in SAS and considered significant at *p* < 0.05.

## 3. Results

### 3.1. Carcass pH

The results of antimicrobial carcass wash on carcass pH over a 24 h period of time are displayed in Table 2. As expected, there was no significant difference (*p* = 0.846) in the Pre-Wash pH of treatments as the antimicrobial wash had not yet been applied. At 30 min Post-Wash, LA treatment displayed a lower pH (6.36; *p* < 0.001) compared to the PAA treatments (6.96); however, LA and CON were not different from each other (*p* = 0.512). However, 24 h Post-Wash pH results displayed no differences between the antimicrobial treatments (*p* = 0.513).

### 3.2. Emulsion Stability and Batter pH

The ES Water, ES Fat, and Batter pH data are shown in Table 3. The main effect of carcass TRT had a significant impact on the ES Water separation (*p* = 0.049), had a strong trend for ES Fat separation (*p* = 0.052), and only slightly approached significance on Batter pH (*p* = 0.132). Frankfurters from CON trim had the lowest ES Water separation, while frankfurters from LA trim had the highest ES Water separation, and frankfurters from PAA trim were intermediate. Similarly, frankfurters from CON trim tended to have less ES Fat separation than frankfurters from PAA or LA, which were not different from each other (*p* = 0.8092).

The main effect of PROC had a significant impact on ES Water separation (*p* < 0.001) and ES Fat separation (*p* < 0.001), and it was approaching significance on batter pH (*p* = 0.090). HTP frankfurters had less ES Water separation and less ES Fat separation, indicating greater emulsion stability. Interactions between TRT × PROC were not found for ES Water separation (*p* = 0.701), ES Fat separations (*p* = 0.151), or Batter pH (*p* = 0.253).

### 3.3. Processing Yield (PY) and Purge Loss (PL)

PY results display no significant difference due to TRT (*p* = 0.640; Table 3); however, there was a significant difference in PY for PROC (*p* = 0.002; Table 3) and no TRT × PROC interaction (*p* = 0.866). LTP frankfurters had a higher PY when compared to HTP frankfurters. As shown in Table 3, PL results exhibited no significant differences due to PROC (*p* = 0.189) or TRT (*p* = 0.351). Additionally, the interaction of PROC × TRT was not significant (*p* = 0.369).

### 3.4. Texture Analysis

The texture analysis means comparison can be found in Table 4. Hardness values were significantly different between the TRT main effects (*p* = 0.010), and there was no difference in hardness when comparing the PROC (*p* = 0.548) or the TRT and PROC interaction (*p* = 0.983). CON frankfurters were harder than LA, while PAA were not different from CON or LA frankfurters. A significant difference was found in springiness between PROC (*p* < 0.001) with HTP having a greater springiness value. No difference was found for springiness due to TRT effects (*p* = 0.792) or the interaction of TRT × PROC (*p* = 0.543). Finally, cohesion analysis displayed no difference in TRT (*p* = 0.237) or PROC (*p* = 0.510), and the interaction of TRT × PROC was (*p* = 0.697).

### 3.5. Sensory Analysis

Sensory traits were analyzed with a trained panelist on an 8-point hedonic scale, and the results can be found in Table 5. Carcass TRT and the interaction of TRT × PROC was not significant for any of the sensory traits (*p* > 0.05; Table 5). Furthermore, no differences were observed due to PROC for flavor intensity (*p* = 0.596). However, differences were observed for PROC, with LTP frankfurters having lower hardness scores (*p* = 0.004), less cohesion scores (*p* = 0.031), greater juiciness scores (*p* < 0.001), and a trend for more detectable off flavor (*p* = 0.062) compared to HTP frankfurters.

### 3.6. Instrumental Color

Frankfurter external and internal CIE L*, a*, and b* values over a 60 d display time are shown in Table 6. As expected, display time impact was significant to color measurements over time (*p* < 0.05); however, no TRT × display time, or PROC × display time interactions were found (*p* > 0.05). In addition, the impact of TRT was not significant for external or internal CIE L*, a*, or b*. However, the main effect of PROC showed external CIE L* and a* values was higher for HTP frankfurters (*p* = 0.001), and a strong trend showed higher b* values for HTP frankfurters as well (*p* = 0.053).

## 4. Discussion

Previous studies have found a similar pattern of carcass pH reduction shortly after antimicrobial wash, which then equilibrated after ≈24 h [9,10]. Ellebracht et al. [9] compared peroxyacetic acid and lactic acid carcass washes and found surface pH reduced to 3.3 after peroxyacetic acid wash and observed a pH reduction to 3.4–3.7 after lactic acid wash treatments, with both returning to pH of ≈5.0 after 24 h of storage. Although our data did not show as drastic of a pH drop, this is likely because Ellebracht et al. [9] studies evaluated the surface pH, while our investigation evaluated the superficial internal pH of beef hindquarter muscles. Our findings were similar to others that reported similar pH values of beef trim treated with lactic acid solution (5.78) and were not significantly changed from the control Kang et al. [10]. Therefore, antimicrobial washes have been found to reduce the surface pH immediately after application; however, our data show that muscle pH is far less impacted by antimicrobial washes, particularly after 24 h, which showed no differences.

Difference in ES Water were one of the few quality measurements traits impacted by TRT observed in this study. LA frankfurters had the most ES Water, indicating a reduction in emulsion stability. These results could have been caused an effect of pH similar to Hamm [2], who reported that too low pH values can cause protein denaturation, and they coincide with differences in which the observed LA carcasses had a lower pH 30 min Post-Wash. However, this pH normalized at 24 h, and there was not a significant difference in Fresh Batter pH. The Fresh Batter pH was mildly trending toward significance (*p* = 0.132) with LA and PAA frankfurter batter at lower pH than CON. It is possible the minor drop in pH may have caused some protein denaturation, but additional analysis would need to examine this phenomenon further.

PROC showed differences in ES Water and ES Fat, with HTP having superior values. This could be expected, as it is known that mechanical action must be used to form an emulsion [18], causing processes at higher temperatures to have greater emulsion stability. Similar results were found in Sutton et al. [6], who concluded endpoint chopping temperatures of 15 °C produced the most stable product in reduced fat, high moisture beef frankfurters. Our results vary from other frankfurter work such as that of Whiting [19], who found having elevated batter temperatures decreases gel strength, and Lee [20], who found emulsion stability decreased when higher chopping temperatures were reached. However, these researchers used pork and beef blended trim, resulting in a significantly different fatty acid profile. Beef is higher in saturated fats, meaning the melting point is significantly higher and will therefore require a higher batter temperature to obtain a stable emulsion. Additionally, similar batter pH results were found in Ambrosiadis et al. [21], who reported initial batter pH values of 6.2–6.3 for meat batters when studying the effects of plant oils in comminuted meat products.

Contrasting from ES values, the PY results found within these data determined PROC was significant, but that LTP frankfurters had a higher PY. Jones and Mandigo [5] found comparable PY results of 93% for temperature ranges of 10 to 16 °C. In contrast, Matulis et al. [3] had a much lower PY with their 22 °C and 28 °C treatments; however, these were produced with beef and pork blends while the present study using only beef.

Instrumental hardness values differed in the present study compared to previous research that reported maximum hardness at batter pH of 6.0 and decreased hardness as batter pH increased above 6.3 [3]. TRT had no differences in Fresh Batter pH, but hardness did vary significantly, with CON frankfurters the hardest, LA frankfurters the softest, and PAA frankfurters as intermediate. This is a similar pattern to the differences in ES Water, which could warrant further investigation.

Instrumental springiness was higher in HTP frankfurters, which could be predicted as the particle size was much smaller in HTP frankfurters, resulting in a firmer gel matrix. This is similar to the findings of Small et al. [22], who observed springiness values to be significantly different when increased particle size and mixing time was analyzed. Finally, the cohesion found in this study was similar to findings of Hensley and Hand [23], who reported no change in cohesion for batters produced at different chopping temperatures of 9, 12 or 15 °C.

Sensory values of hardness, cohesion, and juiciness displayed significant differences due to the main effect of PROC; however, no significant difference was found for any sensory traits due to TRT or the interaction of PROC × TRT. The LTP frankfurters have larger lean and fat particle sizes when compared to the HTP frankfurters. The panelist’s scores reflected the LTP frankfurter sensory attributes to be softer, less cohesive, and juicier scores compared to HTP frankfurters. These findings are similar to Matulis et al. [3], who observed softer frankfurters with a reduction in protein–protein interactions of a meat matrix. The LTP frankfurters could have experienced less protein extraction due to the different mechanical action causing the softer texture frankfurter. Moreover, Lee et al. [20] observed that chopping time directly affects hardness and panelist desirability for frankfurters, with shorter chopping time resulting in larger fat globules [24]. Although the frankfurters in this study were chopped at similar time parameters, different mechanical action was used for the PROC groups, causing the LTP groups to have a more desirable texture, juiciness and hardness scoring.

External CIE L*, a*, and b* was not impacted by TRT or TRT × PROC interactions. However, PROC differences showed consistently greater values for HTP frankfurters for all color measurements, indicating HTP frankfurters were darker and redder than LTP frankfurters. Comparative results were found by Small et al. [22], who found that as particle size increased, b* values were significantly different when studying the effects of particle size and sensory characteristics of low-fat, high moisture pork frankfurters.

The main effect of TRT had no effect on internal CIE L*, a*, and b* color values over time, and there was no interaction of TRT × PROC. Similar to the external color findings, PROC differences displayed elevated values for CIE L* and b* but reduced a* values for HTP frankfurters. There is little research about antimicrobial washes affecting the color of processed meat products. However antimicrobial ingredient compounds have been added to frankfurter type sausage and have shown natural extracts of green teas; stinging nettle and olive leaves showed that the L* and a* values decreased after 45 days of storage [25]. Additions of sodium lactate to beef bologna-type sausage reported an increase in fade appearance and at 6 weeks of display [26].

These data indicate that antimicrobial carcass washes had little significant impact on frankfurter quality, while the processing technique was impactful to several frankfurter quality characteristics. This indicates that processors can impact frankfurter composition via processing techniques without concern of antimicrobial washes influencing frankfurter quality.

## Figures and Tables

**Table 1:** Beef frankfurter formulation for high temperature and low temperature processing conditions.

Ingredient	Percent of Batter Formulation (%)
Beef 90% Lean Trim	53.16
Beef Fat	17.72
Water	17.81
Salt	2.03
Sodium Nitrite (6.25%)	0.18
Corn Syrup Solids	2.96
Food Starch	1.48
Vinegar	1.48
Dextrose	0.99
Spice Blend	1.72
Sodium Phosphate	0.44
Sodium Erythorbate	0.04

**Table 2:** Effect of carcass antimicrobial wash on pH of beef biceps femoris.

Sample Time ^2^	Antimicrobial Carcass Treatment ^1^			SEM	Significance of *p*-Value
	CON	LA	PAA		
Pre-Wash	6.88	6.88	6.90	0.11	0.846
30 min Post-Wash	6.74 ^ab^	6.36 ^b^	6.96 ^a^	0.10	<0.001
24 h Post-Wash	5.68	5.75	5.75	0.05	0.513

^1^ CON = 82 °C water, LA = 5% lactic acid wash, PAA = 350 ppm peroxyacetic acid wash. ^2^ Pre-Wash = prior to application of antimicrobial treatment, 30 min Post-Wash = 30 min after application of antimicrobial treatment, 24 h Post-Wash = 24 h after application of antimicrobial treatment. ^ab^ Means lacking a common superscript differ due to the antimicrobial treatment (*p* < 0.05).

**Table 3:** Effects of antimicrobial carcass treatment and processing technique on emulsion stability, fresh batter pH, processing yield, and purge loss of frankfurters.

Trait	Antimicrobial Carcass Treatment ^1^			SEM	Significance of *p*-Value	Processing Technique ^2^		SEM	Significance of *p*-Value
	CON	LA	PAA			LTP	HTP		
ES Water (%) ^3^	2.32 ^b^	2.85 ^a^	2.46 ^ab^	0.14	0.049	3.33 ^a^	1.76 ^b^	0.20	<0.001
ES Fat (%) ^4^	0.58	0.95	0.91	0.11	0.052	1.33 ^a^	0.30 ^b^	0.09	<0.001
Fresh Batter pH	6.38	6.19	6.21	0.07	0.132	6.19	6.33	0.06	0.090
PY (%) ^5^	93.8	93.5	93.9	1.53	0.640	94.4 ^a^	93.1 ^b^	1.52	0.002
PL (%) ^6^	1.3	1.7	2.4	0.52	0.351	2.2	1.4	0.42	0.189

^1^ CON = 82 °C water, LA = 5% lactic acid wash, PAA = 350 ppm peroxyacetic acid wash. ^2^ LTP = 4 °C final batter temperature, HTP = 21 °C final batter temperature. ^3^ Emulsion stability water separation = (mL Expelled Water/Fresh Sample Weight) × 100. ^4^ Emulsion stability fat separation = (mL Expelled Fat/Fresh Sample Weight) × 100. ^5^ Processing Yield = (Final Cooked Weight/Raw Weight) × 100. ^6^ Purge Loss = (Total Package Weight − Frankfurter Weight − Bag Weight)/Total Package Weight × 100. ^ab^ Means lacking a common superscript differ due to the main effect of antimicrobial treatment or processing technique (*p* < 0.05).

**Table 4:** Effects of antimicrobial carcass treatment and processing technique on instrumental texture analysis of frankfurters.

Trait	Antimicrobial Carcass Treatment ^1^			SEM	Significance of *p*-Value	Processing Technique ^2^		SEM	Significance of *p*-Value
	CON	LA	PAA			LTP	HTP		
Hardness (g) ^3^	12,188 ^a^	10,639 ^b^	11,639 ^ab^	341.41	0.010	11,396	11,608	278.76	0.548
Cohesiveness (%) ^4^	33.72	34.40	34.80	0.44	0.237	34.48	34.13	0.36	0.510
Springiness (%) ^5^	0.65	0.65	0.65	0.01	0.792	0.64 ^a^	0.67 ^b^	0.004	<0.001

^1^ CON = 82 °C water, LA = 5% lactic acid wash, PAA = 350 ppm peroxyacetic acid wash. ^2^ LTP = 4 °C final batter temperature, HTP = 21 °C final batter temperature. ^3^ Hardness = peak force during the first compression of the sample, expressed in grams. ^4^ Cohesiveness = (area of work during a second compression/area of work during the first compression) × 100. ^5^ Springiness = sample height during the second compression/original sample height) × 100. ^ab^ Means lacking a common superscript differ due to the main effect of antimicrobial treatment or processing technique (*p* < 0.05).

**Table 5:** Effects of antimicrobial carcass treatment and processing technique on sensory analysis of frankfurters.

Trait	Antimicrobial Carcass Treatment ^1^			SEM	Significance of *p*-Value	Processing Technique ^2^		SEM	Significance of *p*-Value
	CON	LA	PAA			LTP	HTP		
Hardness ^3^	4.47	4.60	4.60	0.15	0.727	4.83 ^a^	4.31 ^b^	0.12	0.004
Cohesion ^4^	4.86	4.73	4.73	0.12	0.678	4.62 ^a^	4.93 ^b^	0.10	0.031
Juiciness ^5^	4.84	5.04	4.84	0.10	0.312	5.30 ^a^	4.50 ^b^	0.09	<0.001
Flavor Intensity ^6^	5.57	5.63	5.44	0.08	0.252	5.57	5.52	0.07	0.596
Off Flavor ^7^	7.31	7.23	7.32	0.08	0.726	7.20	7.38	0.07	0.062

^1^ CON = 82 °C water, LA = 5% lactic acid wash, PAA = 350 ppm peroxyacetic acid wash. ^2^ LTP = 4 °C final batter temperature, HTP = 21 °C final batter temperature. ^3^ Hardness: 1 = Extremely hard, 8 = Extremely soft. ^4^ Cohesion: 1 = Dissolves extremely fast, 8 = Extremely cohesive. ^5^ Juiciness: 1 = Extremely dry, 8 = Extremely juicy. ^6^ Flavor Intensity: 1 = Extremely bland, 8 = Extremely flavorful. ^7^ Off Flavor: 1 = Extremely intense, 8 = Non-detectable. ^ab^ Means lacking a common superscript differ due to the main effect of antimicrobial treatment or processing technique (*p* < 0.05).

**Table 6:** Effects of antimicrobial carcass treatment and processing technique on C instrumental attributes (CIE L* (lightness), CIE a* (redness), and CIE b* (yellowness)) of external and internal surfaces of frankfurters.

External Color ^4^	Antimicrobial Carcass Treatment ^1^				SEM	Significance of *p*-Value	Processing Technique ^2^		SEM	Significance of *p*-Value
	D ^3^	CON	LA	PAA			LTP	HTP		
CIE L*	0	44.08	44.65	44.44	0.551	0.721	41.62 ^a^	47.16 ^b^	0.45	<0.001
	30	44.12	44.13	43.77			43.25 ^a^	46.76 ^b^		
	60	44.31	44.80	45.05			41.72 ^a^	47.72 ^b^		
CIE a*	0	19.28	19.05	18.95	0.513	0.446	18.51	19.68	0.419	0.053
	30	17.87	17.51	18.41			18.24	17.62		
	60	13.33	12.34	12.12			11.86	13.33		
CIE b*	0	26.91	27.68	26.51	0.756	0.703	23.99 ^a^	30.09 ^b^	0.617	<0.001
	30	26.78	26.60	27.85			23.39 ^a^	30.59 ^b^		
	60	25.77	26.66	26.27			22.28 ^a^	30.19 ^b^		
**Internal Color ^5^**										
CIE L*	0	55.30	56.85	56.20	0.607	0.257	51.19 ^a^	61.04 ^b^	0.496	<0.001
	30	56.30	56.88	56.51			51.34 ^a^	61.78 ^b^		
	60	56.03	56.35	56.25			50.59 ^a^	61.83 ^b^		
CIE a*	0	13.69	12.87	13.20	0.267	0.070	13.58 ^a^	12.93 ^b^	0.218	<0.001
	30	13.32	12.98	13.11			13.45 ^a^	12.82 ^b^		
	60	13.08	12.74	12.72			13.22 ^a^	12.47 ^b^		
CIE b*	0	12.01	11.36	11.86	0.610	0.931	9.74 ^a^	13.74 ^b^	0.498	<0.001
	30	10.63	10.74	10.82			9.29 ^a^	12.17 ^b^		
	60	10.85	10.99	10.95			9.46 ^a^	12.39 ^b^		

^1^ CON = 82 °C water, LA = 5% lactic acid wash, PAA = 350 ppm peroxyacetic acid wash. ^2^ LTP = 4 °C final batter temperature, HTP = 21 °C final batter temperature. ^3^ Display period in days. ^4^ External color measurements taken from the surface of two randomly selected frankfurters in triplicate. ^5^ Internal color measurements taken in triplicate from the cut surface of two frankfurters cut lengthwise. ^ab^ Means lacking a common superscript differ due to the main effect of antimicrobial treatment or processing technique (*p* < 0.05).

## Data Availability

Data is contained within the article.
